# Leukemia cutis and all-trans retinoic acid–induced myocarditis in acute promyelocytic leukemia

**DOI:** 10.46989/001c.158170

**Published:** 2026-03-26

**Authors:** Kian J. Rahbari, Parth C. Patel, Sarah Profitt, Justin T. Kelley, Katie A. O’Connell, Anna K. Dewan, Kathryn E. Kennedy, Erica E. Mittlebeeler, Ashwin Kishtagari, Somedeb Ball, Sanjay R. Mohan, Kateryna Fedorov

**Affiliations:** 1 Department of Internal Medicine, Vanderbilt University Medical Center, Nashville, TN, USA; 2 Department of Internal Medicine, Division of Hematology and Medical Oncology, Vanderbilt University Medical Center, Nashville, TN, USA; 3 Department of Pharmacy, Vanderbilt University Medical Center, Nashville, TN, USA; 4 Department of Pathology, Microbiology, and Immunology, Vanderbilt University Medical Center, Nashville, TN, USA; 5 Department of Dermatology, Vanderbilt University Medical Center, Nashville, TN, USA

**Keywords:** Acute promyelocytic leukemia, ATRA-induced myocarditis, leukemia cutis, acute myeloid leukemia

## Abstract

Acute promyelocytic leukemia (APL) is frequently curable in the modern era using the chemotherapy-free regimen of all-trans retinoic acid (ATRA) and arsenic trioxide (ATO). However, rare disease manifestations and treatment complications may threaten these outcomes by requiring intensification or abbreviation of therapy. We present a unique case of a 36-year-old male with newly diagnosed low-risk APL with biopsy-confirmed leukemia cutis and isolated ATRA-associated myocarditis during induction therapy. Both APL leukemia cutis and ATRA-associated myocarditis are exceedingly rare, with each having less than 50 published cases to date. This report offers a comprehensive review of the literature, underscoring the importance of a comprehensive diagnostic evaluation and individualized care to ensure outstanding long-term outcomes for patients with APL.

## Introduction

Acute promyelocytic leukemia (APL) represents up to 10% of all acute myeloid leukemia (AML) cases, predominantly affecting young adults, with a median age at diagnosis of 40. Remarkable advances in treatment have made APL highly treatable and often curable with a chemotherapy-free approach.^1^ Unlike other AML subtypes, risk-stratification of APL relies solely on the total white blood cell count (WBC) at diagnosis. Imaging or cerebrospinal fluid evaluation is guided by patients’ symptoms, as extramedullary disease (EMD) in newly diagnosed APL is exceedingly rare. For low-risk APL (WBC < 10x10^9^/L), treatment with all-trans retinoic acid (ATRA) and arsenic trioxide (ATO) yields excellent long-term outcomes. Differentiation syndrome (DS) is a common adverse event associated with ATRA and can be characterized by leukocytosis, fevers, hypotension, volume overload, pulmonary infiltrates, pleuropericardial effusions, and renal failure.^2^ If recognized early, DS can be managed effectively with glucocorticoids and cytoreductive agents such as hydroxyurea if concurrent leukocytosis is present.^2^ While other ATRA-ATO complications are rare, treatment-emergent adverse events may be misattributed to other causes. Herein, we present a case of a patient with newly diagnosed APL and biopsy-confirmed leukemia cutis who was successfully treated with ATRA-ATO despite developing isolated ATRA-associated myocarditis.

## Case

A 36-year-old male presented for evaluation of progressive fatigue, bruising, and mucocutaneous bleeding. His physical exam was notable for a small violaceous plaque with central eschar on the dorsum of the right hand. Interestingly, his social history included training special forces’ canines using live rabbits. He was found to have pancytopenia (WBC 4.9x10^9^/L, absolute neutrophils 0.6x10^9^/L, hemoglobin 8.2 g/dL, platelets 5x10^9^/L), with 4.3% promyelocytes and 33.6% circulating blasts, concerning for APL. The patient was promptly started on ATRA.

Bone marrow biopsy showed hypercellularity (>95%) with 49% abnormal promyelocytes (bright CD33+, partial dim CD34+, dim CD45+, CD117+, HLA-DR-). t(15;17)​/​*PML*::​*RARA* FISH was positive in 33.5% cells, confirming low-risk APL. Due to the patient’s history of rabbit handling and concern for a zoonotic skin infection, a punch biopsy of the right-hand lesion was performed, revealing an infiltrate of large, myeloperoxidase-positive cells morphologically identified as promyelocytes. Positive t(15;17)/*PML*::*RARA* FISH on this tissue sample confirmed cutaneous APL (Figure 1).

**Figure:**
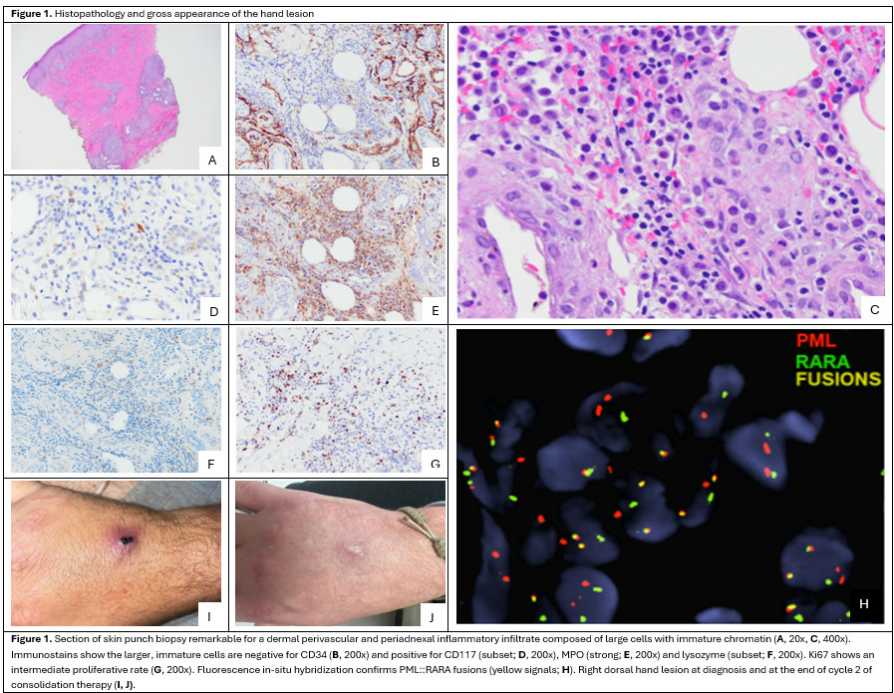


The patient was started on ATRA and ATO induction, along with prednisone 50mg (0.5 mg/kg) daily for DS prophylaxis. The patient developed leukocytosis to 12.9x10^9^/L on day 6, so hydroxyurea was added for further prophylaxis against DS from days 6-15 . A prednisone taper was initiated on day 24.

Due to the presence of EMD at diagnosis, a lumbar puncture was performed on day 26 and was negative for CNS involvement. Also on day 26 of induction, the patient developed chest tightness and pain, nonspecific T-wave inversions on electrocardiogram, and a troponin-I elevation to 2.46 ng/dL. Given concern for therapy-induced myocarditis, ATRA was withheld, and prednisone was escalated back to 40 mg daily, resulting in resolution of chest pain. Transthoracic echocardiogram showed normal ejection fraction, chamber size, and wall motion. Regadenoson stress MRI showed no vasodilator-induced ischemia but did reveal a large area of subepicardial gadolinium enhancement consistent with active inflammation. Cardiac PET/CT revealed diffusely increased uptake in the left ventricular myocardium, highest in the lateral wall, consistent with myocarditis.

A bone marrow biopsy performed on day 28 showed no morphologic evidence of leukemia, and t(15;17)/​*PML*::​*RARA* FISH was negative. The right-hand skin lesion was nearly healed, leaving behind a depressed pink plaque with central heme crust consistent with healing scar. Due to laboratory normalization and continued symptom resolution, the patient was discharged on an extended prednisone taper and off ATRA on day 35.

Upon discharge, the patient abruptly discontinued prednisone due to insomnia, without recurrence of chest pains, despite not receiving any additional myocarditis-directed therapy. ATRA-ATO consolidation began on day 60, 34 days after chest pain onset. The patient has since received three cycles of ATRA-ATO consolidation at standard doses without chest pain recurrence. The hand lesion has fully healed. At the time of writing, the patient remains in MRD-negative remission by reverse transcription quantitative real-time polymerase chain reaction (RT-qPCR) at 16 months from diagnosis, and he remains without any new skin lesions.

## Discussion

While APL has become a largely curable disease, with its non-intensive induction regimen allowing successful treatment outside of specialized academic centers,^3^ unique disease characteristics and rare complications can still compromise long-term outcomes. APL cutis and ATRA-induced myocarditis highlight such challenges, presenting a complex dilemma when encountered in the same patient.

Extramedullary involvement of APL is rare, typically affecting the CNS and skin. The true incidence of APL cutis remains poorly defined, with fewer than 50 reported cases (Table 1).^4–9^ The appearance of skin lesions can vary dramatically from small, isolated macules, papules, or nodules to larger infiltrated plaques.^6^ Although APL cutis has been documented at diagnosis, most cases previously described have occurred in patients with relapsed or refractory disease and have been associated with poor prognosis. Theories suggest APL cutis may arise at sites of vascular disruption, such as bone marrow biopsy sites and intravenous access sites, though this is not typically seen in patients with APL-related hemorrhages.^7^ Interestingly, our patient believed his hand lesion resulted from trauma while handling a rabbit.

**Figure:**
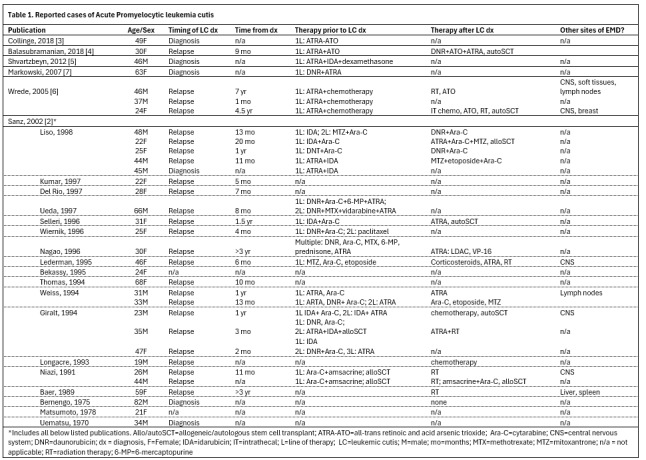


With the introduction of ATRA in the early 1990s, concerns have emerged whether prior ATRA therapy increases the risk of developing EMD. ATRA is associated with myeloid differentiation and has been shown to induce expression of adhesion molecules such as CD11c, CD13, and CD56, facilitating infiltration of extramedullary sites.^9^ However, retrospective analysis of several large studies of patients treated with chemotherapy alone and ATRA-containing regimens did not reveal increased incidence of EMD after ATRA therapy.^9^

The optimal therapy for APL cutis remains unclear. While incorporating cytotoxic chemotherapy into an ATRA-ATO induction backbone is appealing, the diagnosis is often confirmed after ATRA-ATO initiation, beyond the point when anthracyclines are typically administered in high-risk APL protocols. Additionally, there are no data to support the use of maintenance therapy following successful induction and consolidation for low-risk APL.

Given the excellent remission rate of 100% and outstanding long-term survival of 80% at 5 years with ATRA-ATO therapy, every effort is made to adhere to the protocol and minimize treatment disruptions.^10^ Nevertheless, hematologic and non-hematologic toxicities often require dose reductions and treatment interruptions. Abbreviated induction therapy is especially concerning for patients with high-risk features, such as extramedullary disease and leukemia cutis.

Myocarditis is a rare complication of ATRA, with its exact etiology still unclear. Some clinicians believe that it represents DS and should be treated as such. In the literature, myocarditis symptoms appear an average of 19 days after therapy initiation (Table 2).^11–16^ Notably, none of the reported patients had DS prior to onset of myocarditis, although nearly half exhibited concurrent DS symptoms at the time of myocarditis diagnosis. The typical onset of classic DS with ATRA is 7-12 days, making the delayed onset of DS and myocarditis at day 19 unusual.^2^ A new decline in ejection fraction has been documented in 9 of 10 reported cases, with all patients regaining normal cardiac function without long-term sequelae. Distinguishing ATRA-associated myocarditis from DS is challenging, due to common features of pleuropericardial effusion, pulmonary infiltrates, hypotension and volume overload. While fever and leukocytosis may suggest the underlying etiology, these signs and symptoms are also non-specific, with only 7 of 13 cases reporting fever, and only 2 of 11 cases reporting leukocytosis at symptom onset.

**Figure:**
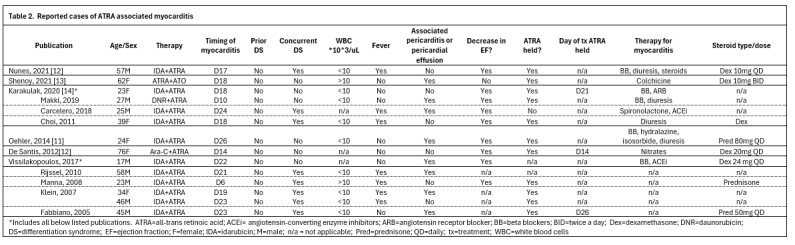


There are no established guidelines for treatment of ATRA-associated myocarditis. In most published cases, ATRA therapy was withheld, and corticosteroids were added, as myocarditis was attributed to severe DS. Treatment for myocarditis itself varied widely in the literature, with patients primarily receiving guideline-directed medical therapy for heart failure with reduced ejection fraction and diuresis.^11,13,14,16^ Lastly, recommendations on when to resume ATRA therapy are lacking. According to our literature review, 10 of 13 patients were able to successfully resume ATRA therapy, which suggests ATRA re-challenge following myocarditis may be feasible.^11–13,15,16^

This case underscores the value of maintaining a high clinical suspicion for diagnosing rare APL manifestations and reinforces that ATRA-induced myocarditis can be reversible with therapy interruption and corticosteroids, without long-term sequelae or need for permanent treatment discontinuation.

### Authorship Contributions

Conceptualization: Kateryna Fedorov, Kian J. Rahbari

Data curation: Kateryna Fedorov, Kian J. Rahbari, Parth C. Patel

Project administration: Kateryna Fedorov, Kian Rahbari

Resources: Justin T. Kelley and Katie O’Connell contributed clinical images for the generation of Figure 1.

Visualization: Kateryna Fedorov, Kian Rahbari, Justin T. Kelley, Katie O’Connell

Writing (original draft): Kian Rahbari, Kateryna Fedorov

Writing (review and editing): all authors

### Competing Interests

No external funding or support was received for this work. The authors declare no competing interests.

### Conflicts of Interest

SP discloses consultancies at Genentech Inc., Rigel Pharmaceuticals Inc., and Eli Lilly & Company outside of this work. AK discloses personal fees from Servier Pharmaceuticals, Incyte, Rigel Pharmaceuticals, Sobi, MorphoSys/Novartis, Geron, and Syndax outside the submitted work.

### Consent to Publish Declaration

Written informed consent for publication was obtained from the patient.

## Data Availability

Not applicable, as this is a case report with no underlying datasets.
